# Expression of Advanced Glycation End-Products on Sun-Exposed and Non-Exposed Cutaneous Sites during the Ageing Process in Humans

**DOI:** 10.1371/journal.pone.0075003

**Published:** 2013-10-07

**Authors:** Maria Crisan, Marian Taulescu, Diana Crisan, Rodica Cosgarea, Alina Parvu, Cornel Cãtoi, Tudor Drugan

**Affiliations:** 1 Iuliu Haţieganu University of Medicine and Pharmacy, Department of Histology, Cluj-Napoca, Romania; 2 University of Agricultural Sciences and Veterinary Medicine, Faculty of Veterinary Medicine, Pathology Department, Cluj-Napoca, Romania; 3 Iuliu Haţieganu University of Medicine and Pharmacy, Clinic of Dermatology, Cluj-Napoca, Romania; 4 Iuliu Haţieganu University of Medicine and Pharmacy, Department of Pathophysiology, Cluj-Napoca, Romania; 5 Iuliu Haţieganu University of Medicine and Pharmacy, Department of Medical Informatics, Cluj-Napoca, Romania; King’s College London, United Kingdom

## Abstract

The glycation process is involved in both the intrinsic (individual, genetic) and extrinsic (ultraviolet light, polution and lifestyle) aging processes, and can be quantified at the epidermal or dermal level by histological, immunohistochemical (IHC), or imagistic methods. Our study is focused on a histological and immunohistological comparison of sun-protected regions versus sun-exposed regions from different age groups of skin phototype III subjects, related to the aging process. Skin samples collected from non-protected and UV protected regions of four experimental groups with different ages, were studied using histology and IHC methods for AGE-CML [N(epsilon)-(carboxymethyl)lysine]. A semi-quantitative assessment of the CML expression in the microvascular endothelium and dermal fibroblasts was performed. The Pearson one-way ANOVA was used to compare data between the groups. In the dermis of sun-exposed skin, the number and the intensity of CML positive cells in both fibroblasts and endothelial cells (*p*<0.05) was higher compared to sun-protected skin, and was significantly increased in older patients. The sun-exposed areas had a more than 10% higher AGE-CML score than the protected areas. No statistically significant correlation was observed between the histological score and the IHC expression of CML. We concluded that in healthy integument, the accumulation of final glycation products increases with age and is amplified by ultraviolet exposure. The study provides new knowledge on differences of AGE-CML between age groups and protected and unprotected areas and emphasizes that endothelium and perivascular area are most affected, justifying combined topical and systemic therapies.

## Introduction

Advanced glycation end products (AGEs) are developed during the Maillard reaction by the glycation and oxidation of different structural proteins [1] and play an important role in age-dependent changes [2]. Glycation involves non-enzymatic reactions between a sugar and a free amine group of Lys and Arg amino acids in proteins. Fructoselysine (FL) is the main product that results from tissue proteins. The extension of the glycation process and its accumulation depend on the glucose concentration and is relatively constant with age [3]. A gradual accumulation of other glycation products occurs in tissue proteins, such as: N-(carboxymethyl) lysine (CML) and N-(carboxymethyl) hydroxylysin (CMhL) that are formed by the oxidative cleavage of FL and pentosidine. Since CML and CMhL involve glycation and free radical oxidation, these products were named “glycoxidation” products. The only products that accumulate with age, CML, CMhl and pentosidine are the result of glycation and oxidative reactions [4], [5]. Protein glycation was described at a cutaneous level [6] but also in organs such as the kidney, blood vessels, and lens. The glycation process is involved in both intrinsic (individual, genetic) and extrinsic (UV light, pollution and lifestyle) aging processes, and can be quantified at epidermal or dermal level by histological, immunohistochemical, imaging or fluorimetric methods [7], [8].

At the cutaneous level, glycation is associated with the aging process and affects cells (endothelial cells, fibroblasts) and structural proteins such as collagen, elastin, glycoproteins, glycosaminoglycans. The dermal extracellular matrix (ECM), modified by glycation, further affects growth, differentiation, motility of the fibroblasts, the cytokine response, enzymatic activity (metalloproteinase) and vascular hemostasis [7]. Both, reactive oxygen intermediates and AGE molecules can induce vascular endothelial growth factor (VEGF) expression, but only AGEs are angiogenic factors [9]. CML represents a general marker of oxidative stress and tissue changes through protein alteration, and plays an important role in the acute and chronic inflammatory process [10]. Moreover, in elderly subjects, the accumulation of AGEs modifies the mechanical properties of human skin through loss of elasticity and increased stiffening [7]. The ECM can be altered by ultraviolet (UVA). Human dermal fibroblasts exposed to UVA in presence of AGEs show decreased cell viability [11]. UVA generates reactive oxygen species (ROS) at the cutaneous level that may accelerate the CML formation and amplify the local degenerative phenomena associated with the ageing process. Moreover, the AGEs accumulation after UVA irradiation was seen in vitro in the dead de-epidermized dermis (DED) [12].

Our study is focused on histological and immunohistological comparisons of sun-protected regions versus sun-exposed regions from different age groups.

## Materials and Methods

### Patients and Study Design

A number of 32 subjects were recruited from the Dermatology Clinic in Cluj-Napoca (Romania). According to their age, the patients were subdivided into four experimental groups (n = 8): group I (under 20 years old), group II (20 to 40 years old), group III (40 to 60 years old), and group IV (over 60 years old). For all subjects skin samples were taken from UV non-protected and UV protected regions. All subjects belonged to skin phototype III.

After local anesthesia using Xylocaine 1%, skin punch biopsies with a 4–5 mm diameter were taken from a sun-protected (left flank area) and sun-exposed region (antero-lateral left arm). Patients had no history of diabetes or other chronic inflammatory diseases.

### Ethics Statement

The study was approved by the Ethical Committee of the University of Medicine and Pharmacy “Iuliu Hatieganu”, Cluj-Napoca (Romania) and an informed written consent was obtained from each of the patients before enrolling them in the study.

### Processing of Tissue Specimens

The skin samples were fixed immediately after excision in 10% neutral buffered formalin for 24 hours and embedded in paraffin wax. Serial sections were cut at 4 µm from each block and stained with hematoxylin and eosin (H&E). The histological appearance of the skin was classified as: Grade 0 (G0), normal skin or minimal perivascular edema and/or pigmentary incontinence; Grade 1 (G1), normal appearance of the dermal matrix but with marked perivascular edema, perivascular mononuclear cell aggregation and/or microvascular basement membrane thickening; Grade 2 (G2), deposition of abnormal matrix proteins (fibrosis with encroachment of collagen into annexes and/or subcutaneous fat, elastosis and/or loss of definition between the reticular and papillary dermis); and Grade 3 (G3), dense uniform dermal fibrosis, epidermal atrophy and annexes loss [13].

### MAb Against AGE-CML and Immunohistochemistry

Serial sections were incubated with primary mouse monoclonal (NF-1G) anti Carboxymethyl Lysine (ab30917; Abcam, Cambridge, UK) diluted in 1% PBS–BSA (bovine serum albumin) at 1/125 at 4°C overnight. The next day, the secondary antibody (labeled streptavidine biotine) was applied, followed by incubation with diaminobenzidine, LSAB System-HRP kit (Code K0679, Dako, Denmark). After washing with distilled water, the slides were counterstained with Mayer haematoxylin for 5 min. Negative controls for each sample were prepared by replacing the primary antibody with mouse IgG1 Negative Control (Code X0931, Dako, Denmark).

### Semiquantitative Grading of CML Immunoreactivity

Immunopositivity for *AGE-CML* was scored separately for both endothelial cells and fibroblasts. The two cell types were differentiated based on their morphology. Only capillary endothelial cells were assessed. Measurements were performed on the five high power fields in the representative sections. The percentage of both immunostained fibroblasts and endothelial cells from the total number of fibroblasts and endothelial cells in each section was assessed in both papillary and reticular dermis. Moreover, the staining intensity of the monoclonal CML antibody was evaluated using a four step-wise system (0, unreactive; +, weak positive; ++, moderately positive; and +++, strongly positive) in accordance with a previous study [14].

The slides were evaluated using an Olympus BX51 microscope with Olympus SP 350 digital camera (Olympus, Tokyo, Japan). “Cell B” basic imaging software (Olympus, Tokio, Japan) was used for semi-automatic counting of immunoreactive cells.

### Statistical Analysis

The data was collected in an Excel worksheet and exported into the SPSS statistical software (IBM Corporation, New York, United States) for analysis. Correlations were assessed with the Pearson correlation coefficient and inter-group mean variation was tested with the ANOVA test.

## Results

### Histological and Immunohistochemical Comparison of Sun-protected Versus Sun- exposed Skin in Different Age Groups

**Figure 1:**
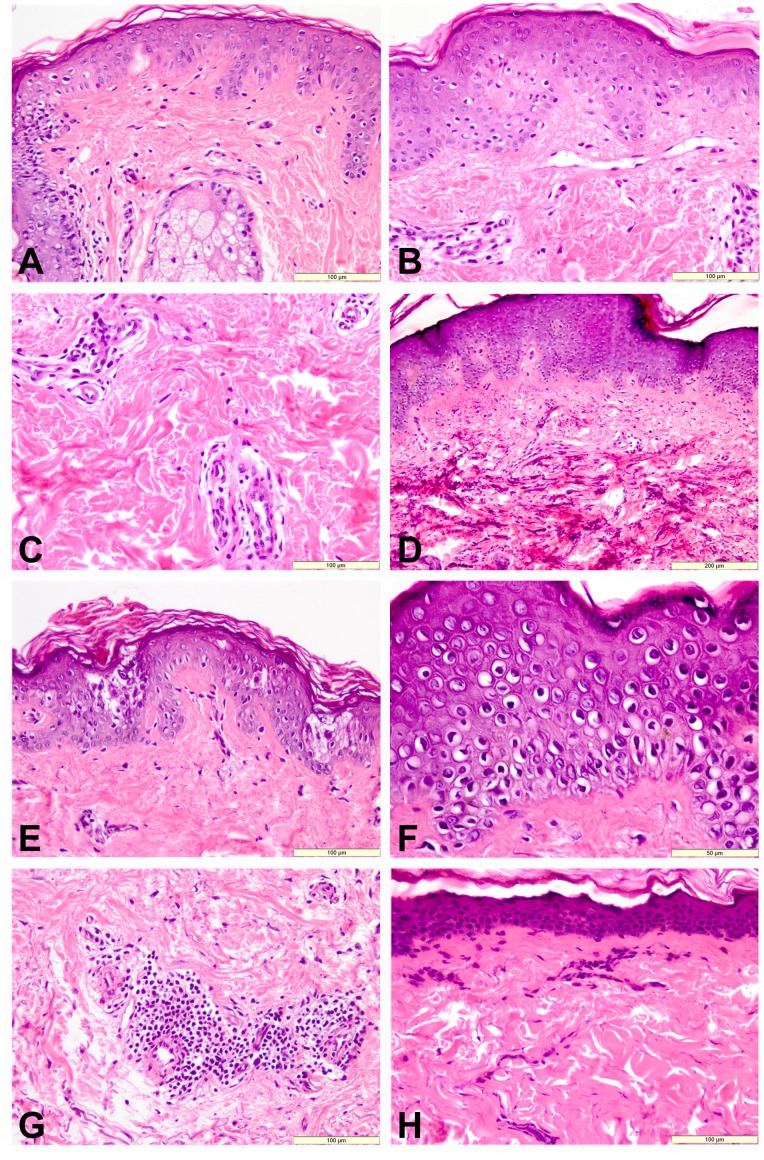
Histological aspects of the skin from different groups and subgroups. **A.** ***Group 1***, UV non-protected regions 1. Intracellular edema and scattered inflammatory cells. HE, bar = 100 µm; **B** and **C.** ***Group 2***, UV non-protected regions; intracellular edema, perivascular edema and mononuclear inflammatory cells, dermal fibrosis. HE, bar = 100 µm; **D** and **E.** ***Group 3*** **,** UV non-protected regions. Intracellular edema, dermal fibrosis and mineralization of collagen fibers; HE, bar = 200 µm; multiple foci of epidermal necrosis. HE, bar = 100 µm; **F** and **G.** ***Group 4***, UV non-protected regions. F - Diffuse intracellular edema of epidermis and multiple apoptotic cells. HE, bar = 100 µm; G - Dermal fibrosis and perivascular mononuclear cell aggregation. HE, bar = 100 µm; **H.** ***Group 4*** **,** UV protected regions. Hyperkeratosis, epidermal atrophy and uniform dermal fibrosis. HE, bar = 100 µm.

In group I (<20 years), the microscope examination revealed edema of the keratinocytes, affecting both basal and spinous layers, perivascular edema and scattered inflammatory cells represented by mononuclear cells into the dermis of the sun-exposed sites (Fig. 1A). No lesions in the protected sites of the first group were observed. In group II (20–40 years), the histological analyses of the sun-exposed regions showed intra- and intercellular edema of the epidermis, perivascular edema, the presence of minimal mononuclear inflammatory cells in the perivascular area (Fig. 1B) and dermal fibrosis (Fig. 1C). At the level of sun-protected sites, only intracellular edema at epidermal level was identified. In group III (40–60 years), multiple foci of epidermal necrosis (Fig. 1E), intracellular edema, several apoptotic cells, uniform dermal fibrosis with mineralization of collagen fibers (Fig. 1D) and skin appendages loss were seen in the sun-exposed areas. In the sun-protected sides the lesions were moderate and characterized by intracellular and perivascular edema, and mononuclear cells infiltrate. In group IV (>60 years), the histological exam of the sun-exposed sites showed advanced lesions such as diffuse epidermal edema and several apoptotic cells (Fig. 1F). In the dermis, solar elastosis, dilation of vessels and microvascular basement membrane thickening, scattered fibroblasts and small aggregates of lymphoid cells in the perivascular area (Fig. 1G) were observed. In contrast to sun-exposed areas, in the protected skin regions, epidermal atrophy (Fig. 1H) and hypocellularity of vascular cells, lymphocytes and fibroblasts, mainly in the upper dermis, dense uniform dermal fibrosis and adnexal loss were present. The score of skin lesions was semiquantified and summarized in Table 1.

**Table 1:** The histological score of skin lesions on different age groups.

Group/years old	Subgroup	No. of Patients	G0^#^	G1	G2	G3
<20 (I)	*UV+	8	6	2	0	0
	**UV–	9	9	0	0	0
20–40 (II)	UV+	9	1	5	3	0
	UV–	8	5	3	0	0
40–60 (III)	UV+	9	0	3	5	1
	UV–	8	0	6	2	0
>60 (IV)	UV+	8	0	1	7	0
	UV–	9	0	1	4	4

* UV+ (UV nonprotected skin); **UV– (UV protected skin); ^#^G0–G3 (Degree of skin lesions).

### Semiquantitative Analysis of Staining for AGE-CML

**Figure 2:**
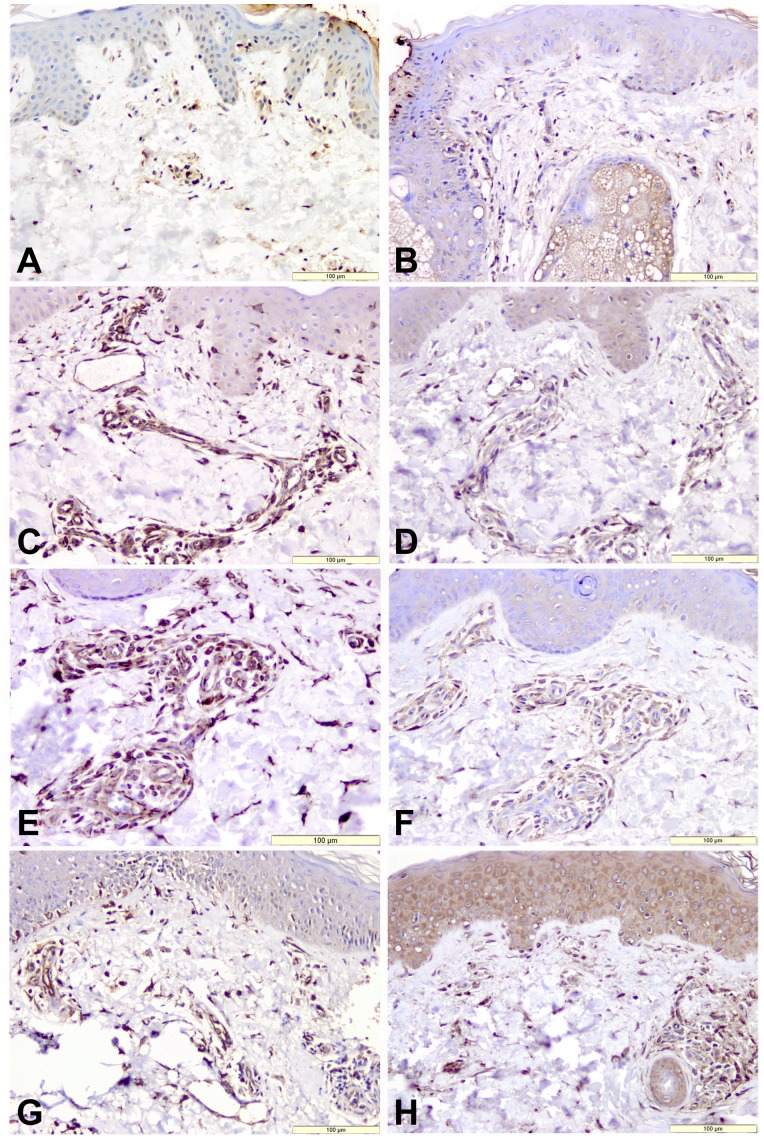
Immunostaining for AGE-CML from UV non-protected areas: group 1(A), group 2 (C), group 3 (E), group 4 (G) and UV protected areas: group 1(B), group 2 (D), group 3 (F), group 4 (H). The presence of AGE-CML expression on fibroblasts, endothelial cells, epidermis and inflammatory cells. Haematoxylin counterstained (bar = 100 µm).

Immunostained products were identified in all patient groups. In both sun-protected and sun-exposed skin areas, the CML expression was predominantly and more intense in the upper dermis than in the deeper dermis, but variably on fibroblasts and endothelial cells (Fig. 2). In all experimental groups, in the dermis of sun-exposed skin (Figure 2 panels A, C, E and G), the number and the intensity of CML positive cells was higher compared to sun-protected skin (Figure 2 panels B, D, F and H). The distribution pattern of the CML - expressing cells was semiquantified and summarized in Table 2.

**Table 2:** Semiquantification of immunohistochemical CML staining.

Group/years old	Subgroup	Basal layer of epidermis	Upper dermis	Deeper dermis	Fibroblasts	Endothelial cells
<20 (I)	*UV+	+	++	+	++	+
	**UV−	−	+	−	+	+
20–40 (II)	UV+	++	+++	+	+++	+++
	UV–	+	+	+	+	+
40–60 (III)	UV+	++	+++	++	+++	+++
	UV–	+	++	+	++	+
>60 (IV)	UV+	++	+++	++	+++	+++
	UV−	++	++	++	++	++

* UV+ (UV nonprotected skin); **UV – (UV protected skin); − nonreactive, + weak, ++ moderate, +++ strong intensity of CML.

### Correlation between the Histological Skin Score and the AGE-CML Expression

Immunopositivity for AGE-CML was scored separately for both endothelial cells and fibroblasts. Measurements were performed on the five high power fields in the representative sections. The percentage of immunostained fibroblasts and endothelial cells are presented in Table 3. There were no statistically significant positive correlations between the histological skin score and the AGE - CML expression neither in fibroblasts or endothelial cells (Table 3).

**Table 3:** The percentage of immunostained fibroblasts and endothelial cells.

Group		Age (years)	AGE-CML %fibroblastsnon-protected	AGE-CML %endothelialcells non-protected	AGE-CML %fibroblastsprotected	AGE-CML % endothelial cells Protected
1	Mean	19.25	29.5450	33.257	24.2350	25.090
	SDV	0.957	2.53795	7.4452	3.67570	2.7753
2	Mean	31.00	37.6375	38.108	32.1725	32.345
	SDV	3.162	1.59903	2.7149	3.22274	3.8365
3	Mean	52.25	47.7800	51.188	42.9050	48.978
	SDV	7.274	3.09702	2.8474	5.13657	2.8584
4	Mean	67.25	62.4475	61.115	57.3400	57.218
	SDV	4.646	5.25768	6.0352	6.28064	8.2066
Total	Mean	42.44	44.3525	45.917	39.1631	40.908
	SDV	19.626	13.0456	12.1652	13.4997	13.9342

SDV = Standard deviation.

**Figure 3:**
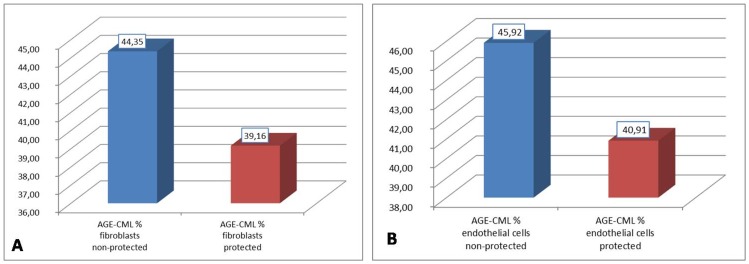
AGE-CML expression in fibroblasts and endothelial cells from both UV protected and non-protected sites. (**A**) AGE-CML is statistically higher in non-protected fibroblasts than in protected tissues (paired t-test probability is less than 0.001); (**B**) AGE-CML is statistically higher in non-protected endothelial cells than in protected tissues (paired t-test probability is less than 0.001).

In order to test the differences between protected and non-protected areas, paired t-tests for both endothelial cells (Fig. 3A) and fibroblasts (Fig. 3B) were performed. There was a significant statistical difference between AGE-CML expression in both fibroblasts and endothelial cells (*p<*0.05) between protected and non-protected samples. The sun-exposed areas had a more than 10% higher AGE-CML score than the protected areas. The percentage of AGE-CML expression in the endothelial cells from sun-exposed sites was 45.92 compared to 40.91 from endothelial cells of the protected areas. Additionally, the average percentage values of the AGE-CML expression in the fibroblasts from sun-exposed areas was 44.35 compared to 39.16 from the fibroblasts of the protected sides.

**Figure 4:**
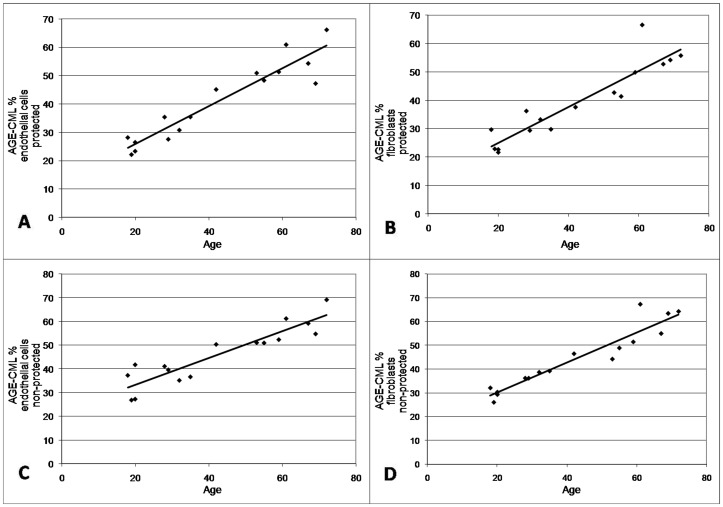
The graphical representation of the correlation analysis between the age of the patients and the AGE-CML score. (**A**) AGE-CML percentage is proportional with the patients’ age in endothelial cells in protected regions (Pearson correlation coefficient = 0.941). (**B**) AGE-CML percentage is proportional with the patients’ age in fibroblasts in protected regions (Pearson correlation coefficient = 0.921). (**C**) AGE-CML percentage is proportional with the patients’ age in endothelial cells in non-protected regions (Pearson correlation coefficient = 0.912). (**D**) AGE-CML percentage is proportional with the patients’ age in fibroblasts in non-protected regions (Pearson correlation coefficient = 0.949).

The probability of the ANOVA test for the assessment of the variation between groups was for all variables less than 0.0001 suggesting a statistical significant difference between the groups. Based on this result, a correlation analysis between the age of the patients and the AGE-CML score was performed. The graphical representation of our results is shown in Figure 4 (A–D).

## Discussion

The integument represents an important model for the study of the aging process, as it is submitted to both intrinsic and extrinsic factors. The various mechanisms, such as the accumulation of genomic mutations, hormonal deprivation, accumulation of toxic metabolites, oxidative damage and glycation, operate simultaneously at skin level, inducing structural, functional and clinical alterations.

Several studies have proven that glycation receptors are present in healthy skin [15]. It is well known that AGE accumulates during natural ageing, but is more prevalent in conditions of oxidative stress (UV exposure, atherosclerosis, neurodegenerative diseases, diabetes mellitus) [10].

The dermis is the main structure affected by glycation, that induces fibroblast activation, increase of different matrix molecules (collagen, elastin), increase of metaloproteinase production (MMP 1, 2 and 9), thickening of the basement membranes, changes of alpha 6 and beta 1 integrin patterns in the epidermis [16].

In this study, we have determined the distribution pattern of AGE-CML in skin biopsies from exposed and non-exposed sites which were collected from patients belonging to different age groups, in order to establish a correlation with the histological score. We also conductd a complete histopathological description to assess the chronic inflammatory changes according to age and protected/unprotected area at the skin phototype III individuals.

Routine histology revealed different aspects that correlate with the age interval and UV exposure. The sequence of histological changes is the result of both structural and functional changes of cells and their impact on the ECM. Similar histological aspects were described by other authors as well [17], [18]. The fibrosis and elastosis processes that become visible early in photoexposed areas and amplify with age and UV exposure, may be identified even by using high frequency ultrasound [8].

Our histological data is in accordance with literature, showing that important structural, biochemical, molecular, architectural and functional dermal changes (including glycation), start before the age of 35, (critical interval), increase rapidly due to intrinsic processes and are amplified by UV. In our opinion, this age threshold represents the best time for the initiating of anti-aging therapies, which must combine products that both stimulate protein synthesis and inhibit the glycation process [8], [12]. Our findings confirm that CML-like immunoreactive material is present in both fibroblasts and endothelial cells from the dermis, and is increased in older patients and in the sun-exposed sides.

Similar reports showed that AGEs bind to fibroblasts cell membranes receptors and interact with intracellular vimentin filaments, with DNA, contributing to the progression of skin aging [19]. CML represents a biomarker of oxidative stress, and is absent in the tissue of young individuals. CML is involved in the aging process, being accumulated in skin collagen, elastic fibers, glycosaminoglycans of normal people [20], but the interactions of AGEs with dermal fibroblasts, especially in the context of skin aging, remain unclear. AGE receptors (RAGE) are also expressed in microvascular endothelial cells [21]. A recent report revealed that RAGE expression was increased in sun-exposed skin and RAGE-positive cells were mainly represented by keratinocytes from basal layer, skin fibroblasts, dermal dendrocytes, endothelial cells and lymphocytes [15].

Our study revealed the CML distribution at epidermal level, especially in the germinative layers, suggesting the impact of cytokines on glycation mechanisms, and implicitly the role of UV in the modulation and accumulation of glyco-oxidation products at dermal level. Keratinocytes are highly active skin cells that release VEGF (vascular endothelial growth factor) and FGF (fibroblast growth factor) leading to endothelial cell activation and enhanced angiogenesis, finally leading to a wound-healing response [22]. Endothelial cells also express MMPs which degrade ECM components thus recruiting circulating macrophages and leukocytes, further secreting different collagenases that can degrade the ECM [23].

CML distribution and expression is modified in microvascular endothelial cells, depending on age and UV exposure [21]. In the present study the sun exposed skin biopsies from older patients revealed an increased inflammatory response with mononuclear cells compared to sun-protected areas and younger patients, but this evidence was not correlated with CML expression.

According to our data, at the cutaneous level glycation is involved in the aging process and affects simultaneously, directly and indirectly, key cells, mostly endothelial cells which are more sensitive to UV rays compared to fibroblasts (see Fig. 4 A–B). No statistically significant correlation was observed between the histological score and the immunohistochemical expression of CML, which proves that cutaneous aging, apart from the glyco-oxidation products, is the result of numerous subtle mechanisms, some of which still remain unclear. Further studies are necessary to identify the mechanisms involved in skin aging and their effects on skin structure, mechanics, and function. Our study opens a new approach perspective of anti-aging therapies.

We concluded that in healthy integument, the accumulation of final glycation products increases with age and is amplified by ultraviolet exposure. The glycation process interferes with the fibroblasts and the vascular endothelium, mostly inducing changes at ECM level. No statistical significant correlation was observed between the histological score and immunohistochemical expression of CML. Approved (FDA) antiaging therapeutic options (topical and systemic) that target glycated molecules exist, thus this research has a translational significance providing new knowledge on differences of AGE-CML between age groups and protected and unprotected areas of skin phenotype III subjects, and that endothelium and perivascular area are most affected, justifying combined topical and systemic therapy.
